# Role of *Anopheles funestus* in sustaining residual malaria transmission in central Senegal

**DOI:** 10.1186/s12936-026-06090-w

**Published:** 2026-08-14

**Authors:** Mouhamadou Bassir Faye, Abdoulaye Kane Dia, Adji Fatou Diouf, Balla Moussa Silamé, Youssouph Coulibaly, Moussa Diop, Seynabou Mocote Diédhiou, Omar Thiaw, Lassana Konaté, El Hadji Amadou Niang, Ibrahima Dia, Badara Samb

**Affiliations:** 1 Laboratoire d’Ecologie Vectorielle et Parasitaire, Université Cheikh Anta Diop de Dakar, Dakar, Senegal; 2 Centre International de Recherche et de Formation en Génomique Appliquée et de Surveillance Sanitaire (CIGASS), Université Cheikh Anta Diop de Dakar, Dakar, Senegal; 3 Pôle de Zoologie Médicale, Institut Pasteur de Dakar (IPD), Dakar, Senegal

**Keywords:** *Anopheles funestus*, Residual malaria transmission, Central Senegal

## Abstract

**Background:**

*Anopheles funestus* is one of the major malaria vectors in sub-Saharan Africa. In Senegal, it is found across nearly all biogeographical areas, often replacing *An. gambiae* s.l. and sustaining malaria transmission during the dry season. Despite its recognized role as a major vector, the specific contribution of *An. funestus* to malaria transmission in many malaria-endemic areas remains underexplored. This study aims to assess the contribution of *An. funestus* in malaria transmission in two health districts in Central Senegal.

**Methods:**

Adult mosquitoes were collected from July 2018 to November 2019 in the health districts of Nioro (Ndramé Ndimb) and Ndoffane (Tawa Mboudaye) using Human Landing Catches (HLCs) and Pyrethrum Spray Catches (PSCs). Anopheline specimens were identified morphologically using standard dichotomous keys, with species confirmation performed by polymerase chain reaction (PCR). *Plasmodium falciparum* infection and blood meals sources were determined using Enzyme-Linked Immunosorbent Assay (ELISA) techniques.

**Results:**

A total of 6,932 *Anopheles* mosquitoes were collected. Of these, 73.7% (5,108/6,932) were identified as *An. funestus*, which was the predominant species at both study sites, with 1,364 specimens collected in Nioro and 3,744 in Ndoffane. Molecular analysis confirmed that *An. funestus* sensu stricto (s.s) was the only member of the *An. funestus* group recorded. In Ndramé Ndimb, the mean human biting rate and indoor resting density were 2.8 bites per person per night (b/p/n) and 9.0 females per room (f/r), respectively, compared to 12.4 b/p/n and 19.1 f/r in Tawa Mboudaye. The *P. falciparum* circumsporozoite rate was 0.3% in both villages, resulting in estimated entomological inoculation rates (EIRs) of 0.008 and 0.038 infected bites per person per night in Ndramé Ndimb and Tawa Mboudaye, respectively. The human blood index was relatively low, with only 22.3% (324/1,452) of *An. funestus* specimens found to have fed on humans.

**Conclusions:**

This study highlights the predominance of *An. funestus* and its important role in sustaining residual malaria transmission in central Senegal, even where overall transmission intensity remains low.

## Background

Malaria remains a major global public health challenge and is the most prevalent parasitic disease worldwide. In 2024, the World Health Organization (WHO) estimated 282 million cases and 610,000 deaths. The African region was disproportionately affected, accounting for 94% of cases and 95% of deaths, with the highest burden observed among children under five years of age and pregnant women [1].

Disruptions to health systems during the COVID-19 pandemic may have contributed to a resurgence of malaria cases in the sub-Saharan region, thereby reversing previous gains [2]. In Senegal, the epidemiological situation between 2015 and 2019 was characterised by an increase of 38% in malaria cases and a reduction of 7% in malaria-related deaths [3].

According to the NMCP, in 2024, Senegal recorded 1,089,921 confirmed malaria cases and 1,325 malaria-related deaths, underscoring the continued public health burden of the disease [4]. Malaria transmission in Senegal is predominantly caused by *P. falciparum*, which accounts for more than 95% of reported cases. Other species such as *P. malariae* and *P. ovale* are occasionally detected, but they contribute only marginally to the overall malaria burden. *Plasmodium vivax* is extremely rare, with sporadic cases mainly linked to imported infections. The predominance of *P. falciparum* is consistent across both urban and rural settings, and this species is responsible for the most severe clinical forms of the disease. Consequently, malaria control strategies in Senegal are primarily designed to target *P. falciparum*, while surveillance systems remain attentive to the presence of the less common species to ensure comprehensive monitoring of parasite diversity [5]. Malaria transmission across the sub-Saharan Africa is driven by various *Anopheles* mosquito species, which exhibit highly heterogeneous transmission dynamics [6–10]. Although the entire region is at risk, infection and disease incidence vary considerably across the continent and even within small geographical areas [8, 11]. According to the National Malaria Control Programme (NMCP), Senegal has a relatively low malaria incidence compared to other Sub-Saharan countries [12]. The transmission intensity is strongly influenced by several entomological factors including diversity, longevity, aggressiveness and high anthropophilic behaviour of vector species, contributing to Africa being the most malaria-affected continent [13].

In sub-Saharan Africa, malaria vectors generally belong to the *An. gambiae* complex and the *An. funestus* group, both being the major targets of vector control interventions. In Senegal, four known sibling species of the *An. gambiae* complex (*An. arabiensis, An. gambiae, An. coluzzii* and *An. melas*) and *An. funestus s.s* are known as main species that play a key role in malaria transmission [14–18].

*Anopheles funestus* is found in various biogeographical zones in Senegal, notably in the Senegal River basin [16] and the Sudanian and Sudano-Guinean zones [14, 19]. Primarily known as a highly anthropophilic and endophilic species, *An. funestus*, however, can display variable feeding behaviour, as observed in areas with abundant livestock or where long-lasting insecticidal nets (LLINs) are massively used. In central-western, south-eastern and southern Senegal, *An. funestus* exhibits high anthropophilic behaviours, whereas in the northern part of the country, it demonstrates a well-known zoophilic trend [19–21].

In Senegal, malaria transmission is generally seasonal, with two peaks: one during the rainy season, driven by *An. gambiae* complex species, and another during the dry season, driven by *An. funestus* [22]. However, in some areas, particularly the Sudano-Sahelian zone, in central Senegal, the contribution of *An. funestus* in malaria transmission remains poorly documented. Entomological surveys conducted in 2016 and 2018 revealed significant malaria prevalence in the districts of Nioro and Ndoffane, coinciding with high densities of *An. funestus* (Data not published). These preliminary findings have prompted the need for further investigations to elucidate the contribution of *An. funestus* in malaria transmission in these settings. This study aims to assess the role of *An. funestus* in malaria transmission in the Nioro and Ndoffane districts, in central Senegal.

## Methods

### Study sites

**Fig. 1:**
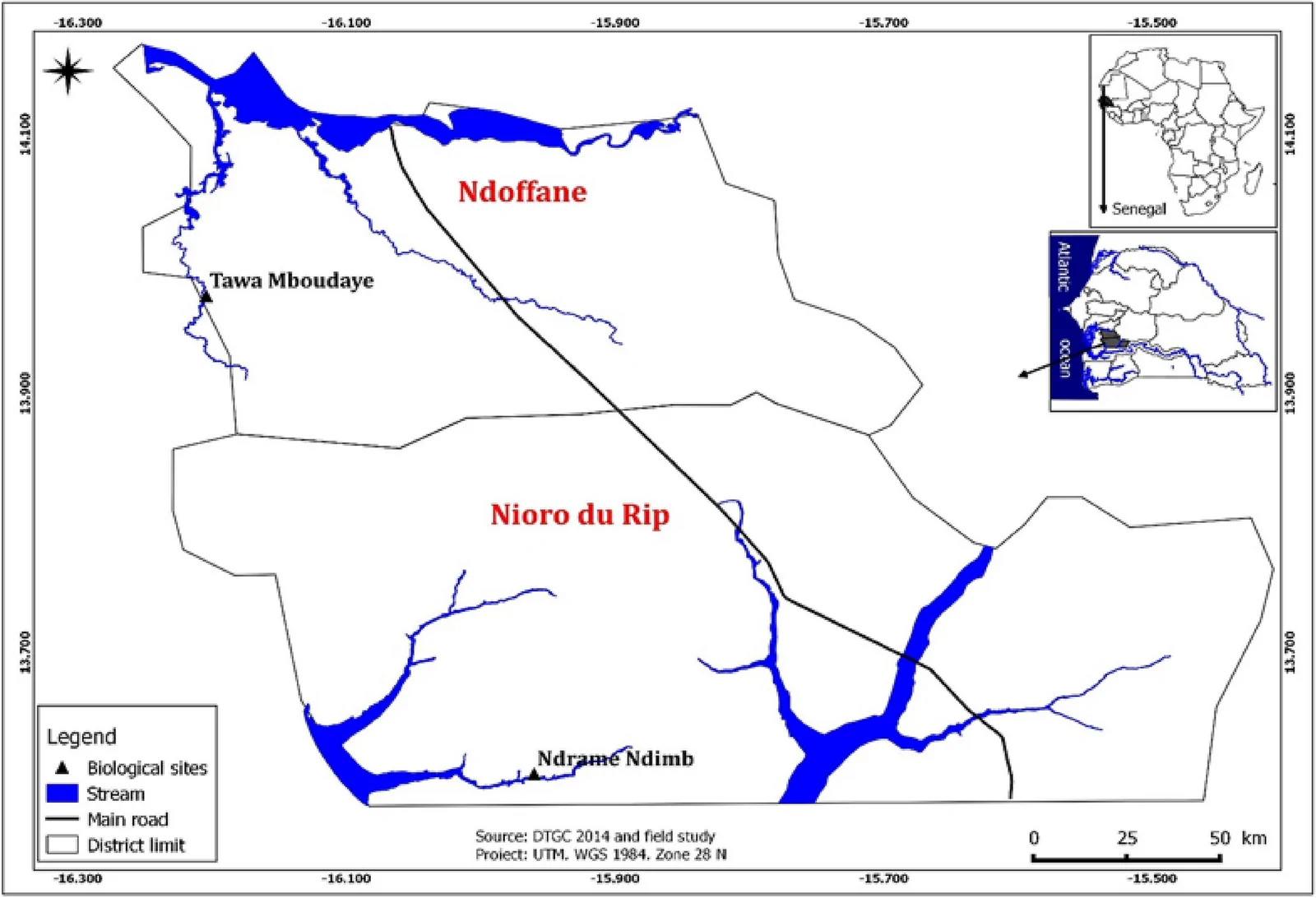
Location of the study sites

The study was carried out from July 2018 to November 2019 in the villages of Ndramé Ndimb (13°36'N, 15°57'W) and Tawa Mboudaye (13°58'N, 16°12'W), respectively located in the Nioro and Ndoffane health districts of Kaolack region, central Senegal in the Sudano-Sahelian zone (Fig. 1). This area is characterized by a short rainy season, from June to October, and a long dry season, from November to May, with annual temperatures ranging between 18 and 40 °C, January being the coldest month and May the warmest one.

Mosquito sampling was conducted monthly from July 2018 to November 2018. Due to logistical and organizational constraints, no collections were performed in December 2018. From January 2019 through November 2019, the sampling frequency was transitioned to a bimonthly schedule (every two months). This adjustment was implemented to ensure long-term monitoring of the *An. funestus* population across multiple seasons while maintaining logistical feasibility.

The two study village differ in housing structure and hydrographical features. Ndramé Ndimb is composed predominantly of traditional dwellings with mud walls and thatched roofs, whereas Tawa Mboudaye features modern housing with cement walls and corrugated iron roofs. Agriculture and livestock farming represent the main economic activities in both sites. Livestock, including small ruminants, cattle, horses and poultry, are maintained throughout the year, with nomadic herding practices primarily taking place during the dry season.

Globally, the vegetation in the study area is typical of a woodland savannah, dominated by grasses, scattered shrubs, and large trees. However, the hydrographic characteristics differ between the two sites and strongly influence the availability of mosquito larval habitats.

Tawa Mboudaye is crossed by a permanent river, which maintains water throughout the year and is bordered by dense emergent vegetation, predominantly *Typha australis*. These permanent, vegetated water bodies provide stable and productive larval habitats, particularly favorable for *An. funestus*, a species known to preferentially breed in permanent or semi-permanent aquatic habitats with emergent vegetation.

In contrast, Ndramé Ndimb is characterized by a floodplain system that forms temporary to semi-permanent pools during and after the rainy season. These pools are also largely covered by *Typha australis* and persist for several months, creating suitable breeding habitats for mosquitoes, including *An. funestus*. The seasonal availability of these larval habitats likely contributes to temporal variations in mosquito density observed at this site.

In terms of vector control, the study area has benefitted from the National Malaria Control Programme’s (NMCP) strategy of Universal Coverage with Long-Lasting Insecticide-Treated Nets (LLINs). A mass distribution campaign was conducted across the Kaolack region shortly before the study began, contributing to the national effort of distributing over 9 million nets. These nets were primarily standard pyrethroid-treated nets (PermaNet® 2.0). While the campaign prioritized vulnerable groups such as pregnant women and children under five years of age, the strategy aimed for at least one net for every two people in every household. These interventions remained the primary tool for reducing human-vector contact in the study villages throughout the 2018–2019 monitoring period.

### Field sampling and processing of mosquitoes

Human Landing Catches (HLC) were conducted by adult volunteers who provided informed oral consent prior to participation. The safety of all participants was ensured through continuous medical supervision during and after the study period. Volunteers were provided with long-lasting insecticidal nets (LLINs) for use during rest periods and had daily access to a medical coordinator (local Head Nurse). Any participant reporting symptoms suggestive of malaria or other febrile illness received immediate, free diagnostic testing and appropriate treatment according to national guidelines. Active clinical follow-up was maintained for 21 days after completion of field activities, and no malaria or arboviral infections were detected among the volunteers.

Adult mosquitoes were captured in each study site using two methods, Human Landing Catch (HLC) to collect host-seeking females, and Pyrethrum Spray Catch (PSC) which targeted indoor resting females. HLCs were conducted in three randomly-selected houses in each village, from 8 p.m. to 6 a.m., by recruiting twelve volunteers for each site, organised into two groups, each of which working alternately in pairs equitably distributed inside and outside of selected houses. The first pair operated between 8 p.m. and 1 a.m., and the second between 1 a.m. and 6 a.m., corresponding to a capture effort of six persons per night at each study site. Captures were performed on two consecutive nights. Any bias due to the differential attractiveness of the capture operators was minimised by alternating the pairs between capture points and time slots. PSCs were conducted in ten randomly-selected dwellings, from 7 a.m. to 9 a.m., the morning following the second night of HLCs. PSC technique consisted of laying white sheets on the floor of selected rooms and spraying inside with an over-activated solution of pyrethroids. After ten minutes, stunned mosquitoes were removed and their abdominal status was specified (i.e. unfed, fed, half-gravid and gravid).

Upon collection, mosquitoes were sorted, morphologically identified to species level using conventional dichotomous keys [22, 23] and counted. A subsample of *An. funestus* from HLC-specimens (host-seeking females) was used for ovary dissection to determine physiological age, as described by Detinova [24]. All *Anopheles* specimens were stored individually in labelled Eppendorf tubes with desiccant for subsequent laboratory analyses.

### Mosquitoes laboratory processing

Subsamples of *An. funestus*, collected by HLC and PSC, were analysed using a Polymerase chain reaction (PCR) to identify sibling species within the *An. funestus* group [25]. For each mosquito, the head and thorax were used for the detection of *Plasmodium falciparum* infection using a circumsporozoite protein enzyme-linked immunosorbent assay (CSP-ELISA), as these body parts contain the salivary glands where sporozoites are localized [26]. The abdomen of blood-fed females was used for blood meal source identification by ELISA, following the method described by Beier et al. [27].

The remaining body parts (legs and wings) were preserved for molecular analyses. Genomic DNA was extracted using the CTAB 2% (Cetyltrimethylammonium bromide) method described by Doyle et al. [28]. Species identification within the *Anopheles funestus* group was performed by PCR following the protocol of Koekemoer et al. [25], using species-specific primers. PCR amplifications were carried out using a 96-well Bio-Rad T100 PCR Thermal cycler under the conditions described in the original protocol.

### Data analysis

Human Biting Rate (HBR) was calculated as the ratio of mosquitoes collected by HLC to the total of person-nights. The parity rate was estimated as the proportion of parous mosquitoes among the total of dissected specimens. Indoor Resting Density (IRD) was determined as the number of mosquitoes per room collected by PSC. The circumsporozoite rate was calculated as the proportion of females infected with *P. falciparum*. The entomological inoculation rate (EIR) was computed as the product of the HBR and the circumsporozoite rate. The human blood index (HBI) was calculated as the proportion of females fed on humans relative to the total identified blood sources, with mixed blood meals counted as multiple sources, with double and triple meals counted twice and three times, respectively.

Statistical analyses were performed using Excel and R software (v4.4.2). Comparison of proportions and means for relevant entomological parameters (endophagic, density, parity and inoculation rates) were performed using appropriate statistical tests. The significance level was set at 5%.

## Results

### Mosquito diversity and abundance

A total of 11,447 mosquitoes from four genera were collected using HLC and PSC (Table 1). *Anopheles* species were predominant (60.6%, n = 6,932), followed by *Culex* spp (36.0%, n = 4,124), *Mansonia* spp (3.4%) and *Aedes* spp (0.01%). Within *Anopheles* genera, *An. funestus* was globally the most abundant species (73.7%; n = 5,108), followed by *An. gambiae* s.l. (25.2% n = 1,747). Other *Anopheles* species were encountered to a lesser extent and included *An. rufipes* (n = 49)*, An. pharoensis* (n = 14), *An. ziemanni* (n = 7), *An. wellcomei* (n = 6), and *An. coustani* (n = 1). In Ndramé Ndimb, *An. funestus* s.l. accounted for 68.2% (1,364/2,001) of collections, followed by *An. gambiae* s.l. which made up 29.2% (584/2,001). In Tawa Mboudaye *An. funestus* s.l. was even more predominant, accounting 75.9% (3,744/4,931) of collections, followed by *An. gambiae* s.l. which represented 23.6% (1,163/4,931). The abundance of *An. funestus* differed significantly between the two villages (Exact binomial test: *p* < 0.001) whatever the collection method (Table 1). On the other hand, the proportion of *An. funestus* s.s was significantly different between the two sites (Exact binomial test: *p* < 0.001). However, no difference was noted in species composition between the two sites.

**Table 1:** Mosquito species composition

Genus/Species	2018				2019			
	Nioro		Ndoffane		Nioro		Ndoffane	
	Ndramé Ndimb		Tawa Mboudaye		Ndramé Ndimb		Tawa Mboudaye	
	PSC	HLC	PSC	HLC	PSC	HLC	PSC	HLC
***Culex spp***	**307**	**547**	**800**	**607**	**430**	**280**	**643**	**510**
***Aedes spp***	**0**	**0**	**0**	**0**	**0**	**0**	**1**	**0**
***Mansonia spp***	**0**	**229**	**0**	**2**	**10**	**149**	**0**	**0**
***Anopheles spp***	**1077**	**385**	**2312**	**1389**	**408**	**131**	**674**	**556**
*An. funestus s.l*	813	306	1694	1196	175	70	409	445
*An. gambiae s.l*	243	72	608	193	224	45	255	107
*An. pharoensis*	1	0	0	0	0	12	0	1
*An. rufipes*	20	0	10	0	9	0	10	0
*An. ziemanni*	0	5	0	0	0	2	0	0
*An. coustani*	0	1	0	0	0	0	0	0
*An. wellcomei*	0	1	0	0	0	2	0	3
**Total**	**1384**	**1161**	**3112**	**1998**	**848**	**560**	**1318**	**1066**
PSC = Pyrethrum Spray Catch; HLC = Human Landing Catch								

The analysis of entomological transmission parameters focused on the *An. funestus* population. Molecular identification confirmed the exclusive presence of *An. funestus* sensu stricto, hereafter referred to as *An. funestus* s.s.

### Indoor resting density of* Anopheles funestus* s.s

The mean indoor resting density (IRD) of *An. funestus* s.s was globally lower in Ndramé Ndimb (9 females per room) compared to Tawa Mboudaye (19.1 f/r) (Wilcoxon rank sum exact test: w = 22, *p* = 0.0104). In Ndramé Ndimb, the highest IRDs were recorded in the dry season, especially in November 2018 (10.4 f/r) and in March 2019 (38.9 f/r). However, in Tawa Mboudaye in 2018, peak densities were recorded at the beginning and at the end of the rainy season, with respectively 54.5 f/r in July 2018 and 23.3 f/r in October. In 2019, highest indoor resting densities were observed both in the rainy season (July) and dry season (March), with IRDs estimated at 15.4 and 20.9 f/r, respectively. Overall, the results indicated that indoor resting densities were higher during the dry season in Ndramé Ndimb, but more so in the rainy season, in Tawa Mboudaye (Table 2).

**Table 2:** Seasonal variation of indoor resting densities of *An. funestus* s.s. collected by pyrethrum spray catch by study sites and collection period

Months		Ndramé Ndimb		Tawa Mboudaye	
	NP	Number	IRD (f/r)	Number	IRD (f/r)
Jul _2018	10	34	3.4	545	54.5
Aug 2018	10	28	2.8	329	32.9
Sept 2018	10	41	4.1	126	12.6
Oct 2018	10	36	3.6	235	23.5
Nov 2018	10	104	10.4	94	9.4
Jan 2019	10	181	18.1	156	15.6
Mar 2019	10	389	38.9	209	20.9
May 2019	10	56	5.6	44	4.4
Jul 2019	10	47	4.7	154	15.4
Sept 2019	10	35	3.5	148	14.8
Nov 2019	10	37	3.7	63	6.3
TOTAL	**110**	**988**	8.9	**2103**	19.1

NP: number of rooms prospected, IRD: indoor resting density, f/r: females per room

### Biting activity of *Anopheles funestus* s.s

**Fig. 2:**
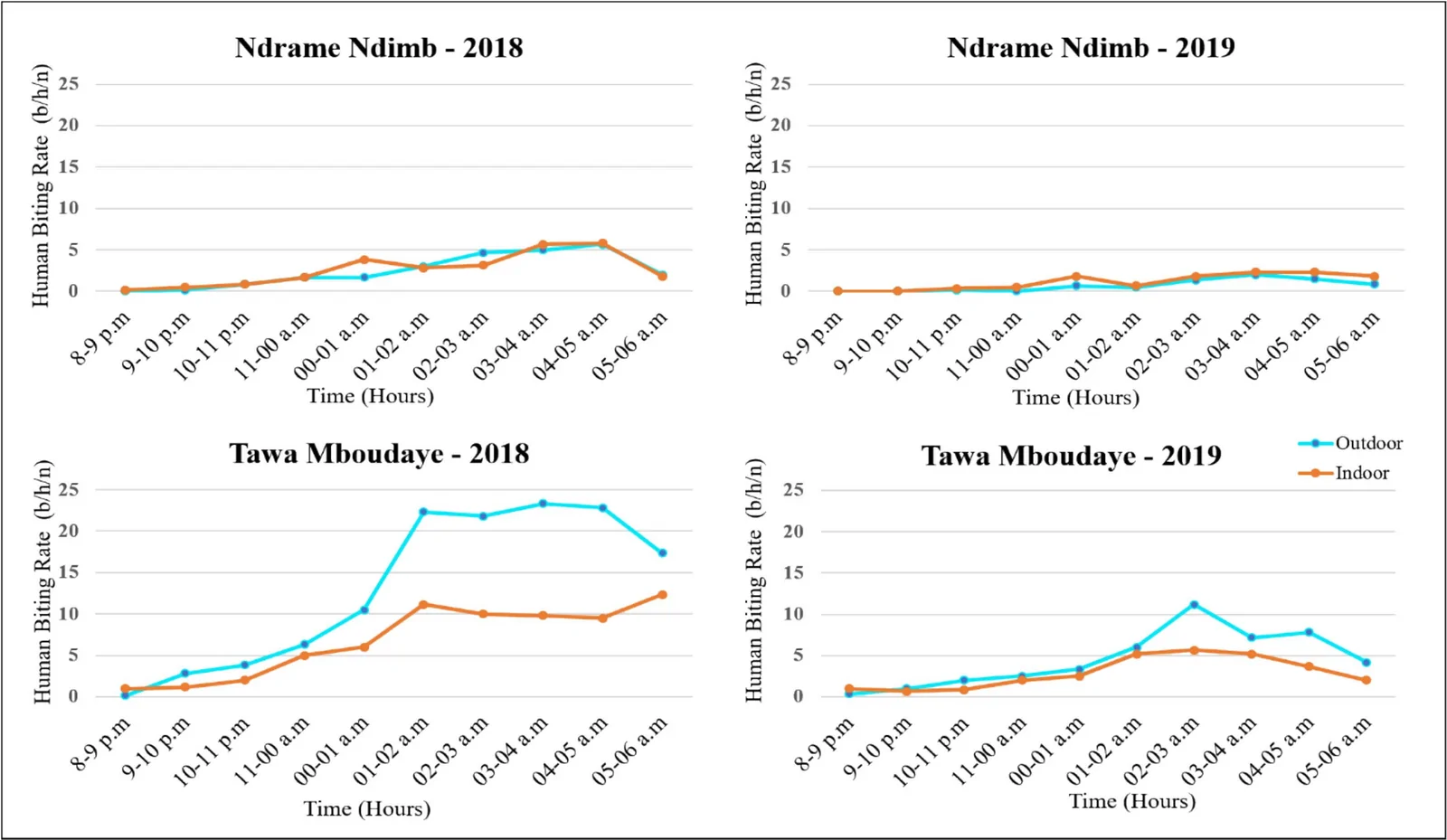
Hourly indoor and outdoor biting activity of *Anopheles funestus* s.s through study sites and collection period

*Anopheles funestus* s.s showed consistent aggressiveness throughout the night both indoors and outdoors. In Tawa Mboudaye, peak biting activity occurred in the second half of the night, specifically from midnight to 1 a.m. in 2018, and between 2 a.m. and 3 a.m. in 2019. In contrast, in Ndramé Ndimb, the peak biting activity was recorded later in the night, between 4 a.m. and 5 a.m. in 2018, whereas two peaks were observed in 2019 between midnight and 1 a.m., and from 3 a.m. to 4 a.m. Overall, biting activity was higher during the second half of the night (from midnight to 6 a.m.) in both Tawa Mboudaye and Ndramé Ndimb (Fig. 2). In Ndramé Ndimb, in 2018, the highest human biting rate (HBR) of *An. funestus* s.s was observed during the rainy season (September), with 5.3 bites per person per night (b/p/n), while in 2019, the highest HBRs were observed both in rainy season (2.2 b/p/n) and dry season (5.8 b/p/n), respectively in September and March. In Tawa Mboudaye, one peak of aggressiveness (27.1 b/p/n) was recorded in 2018, during the rainy season (August), and in 2019, two peaks were observed all year round, the first (17.3 b/p/n) being recorded in rainy season (September), and the second (18.9 b/p/n) in the dry season (March). In the other hand, *An. funestus* s.s displayed various biting behaviour patterns throughout the study site (Table 3). In Ndramé Ndimb, there was no difference between indoor and outdoor biting rates (Exact binomial test: *p* = 0.8771). However, in Tawa Mboudaye, outdoor biting activity was more important (Exact binomial test: *p* < 0.001).

**Table 3:** Seasonal variation of human biting and endophagic rates of *An. funestus* s.s females by study sites and collection period

		Ndramé Ndimb				Tawa Mboudaye			
Mois	HN	Caught	HBR	Indoor	Endo (%)	Caught	HBR	Indoor	Endo (%)
Jul_2018	12	24	2.0	10	41.7	146	12.2	72	49.3
Aug_2018	12	47	3.9	14	29.8	325	27.1	107	32.9
Sept_2018	12	63	5.3	30	47.6	178	14.8	39	21.9
Oct_2018	12	46	3.8	21	45.7	139	11.6	59	42.4
Nov_2018	12	26	2.2	15	57.7	80	6.7	19	23.8
Jan_2019	12	31	2.6	20	64.5	101	8.4	63	62.4
Mar_2019	12	69	5.8	48	69.6	227	18.9	49	24.6
May_2019	12	23	1.9	9	39.9	33	2.8	8	24.2
Jul_2019	12	11	0.9	6	54.5	137	11.4	66	48.2
Sept_2019	12	26	2.2	11	42.3	207	17.3	51	24.6
Nov_2019	12	10	0.8	2	20	68	5.7	47	69.1
**Total**	**132**	**376**	2.8	**186**	**49.5**	**1641**	12.4	**580**	**35.3**

HN = human night, HBR = human biting rate, Endo = endophagic

### Blood meal sources and human blood index

Among the 1,658 blood meals analysed, 1,452 were successfully identified for the four potential vertebrate hosts tested: human, bovine, ovine and horse. A significant difference in host feeding patterns was observed between Tawa Mboudaye and Ndramé Ndimb (Wilcoxon rank sum test: w = 2, *p* = 0.000232). (Table 4). In Ndramé Ndimb, most of blood meals were taken from animals, including horses (55.4%), sheep (5.5%) and bovine (4.34%). Few mosquitoes have fed on humans (11%). Mixed blood meals, indicating feeding on at least two different vertebrates’ hosts were detected in 23.8% of the females tested. A similar host feeding behaviour was observed in Tawa Mboudaye, where horses (36.7%) were the primary host, followed by humans (27.2%), bovine (7.1%) and, sheep (3.6%), in addition to mixed blood meals that made up 25.3%. Overall, the results highlighted low anthropophilic rates in *An. funestus* s.s populations which rather showed a preferential tendency to feed on livestock (Table 4).

**Table 4:** Analysis of blood meal source of *Anopheles funestus* s.s females collected by pyrethrum spray catch

Ndramé Ndimb											
Months	**Human**	**Bovine**	**Sheep**	**Horse**	**H-A**	**A-A**	**Negative**	**Identified**	**Tested**	**HBI (%)**	**Zoophilic rate (%)**
July_2018	0	2	0	15	0	4	2	25	27	0.0	100.0
Aug_2018	1	0	1	10	0	3	0	18	18	5.6	94.4
Sept_2018	0	1	2	18	0	1	1	23	24	0.0	100.0
Oct_2018	1	0	1	22	0	0	2	24	26	4.2	95.8
Nov_2018	1	0	2	30	2	4	4	45	49	6.7	93.3
Jan_2019	10	6	5	30	0	3	18	57	75	17.5	82.5
Marc_2019	10	9	10	67	14	19	24	162	186	14.8	85.2
May_2019	4	1	1	11	0	2	4	21	25	19.0	81.0
July_2019	0	0	1	13	0	4	1	22	23	0.0	100.0
Sept_2019	2	0	0	14	1	2	4	22	26	13.6	86.4
Nov_2019	1	0	1	12	1	1	4	18	22	11.1	88.9
**Sub-Total 1**	**30**	**19**	**24**	**242**	**18**	**43**	**64**	**437**	**501**	**11.0**	**89.0**
Tawa Mboudaye											
July_2018	80	21	11	117	24	51	48	379	427	27.4	72.6
Aug_2018	23	6	8	59	8	14	12	140	152	22.1	77.9
Sept_2018	21	4	2	28	6	6	4	79	83	34.2	65.8
Oct_2018	27	8	0	41	9	3	25	100	125	36.0	64.0
Nov_2018	13	8	0	11	0	1	4	34	38	38.2	61.8
Jan_2019	7	1	3	17	1	5	7	40	47	20.0	80.0
Marc_2019	13	6	5	20	0	2	16	48	64	27.1	72.9
May_2019	9	3	1	8	1	4	1	31	32	32.3	67.7
July_2019	13	3	4	35	4	4	4	71	75	23.9	76.1
Sept_2019	2	11	1	28	6	3	4	60	64	13.3	86.7
Nov_2019	5	1	2	9	4	4	2	33	35	27.3	72.7
**Sub-Total 2**	**213**	**72**	**37**	**33**	**63**	**97**	**127**	**1015**	**1142**	**27.2**	**72.8**
**Overall**	**243**	**91**	**61**	**615**	**81**	**140**	**191**	**1452**	**1643**	**22.3**	**77.7**
H-A: Human and Animal; A-A: Animal-Animal; HBI: Human blood index											

### Parity rate

**Fig. 3:**
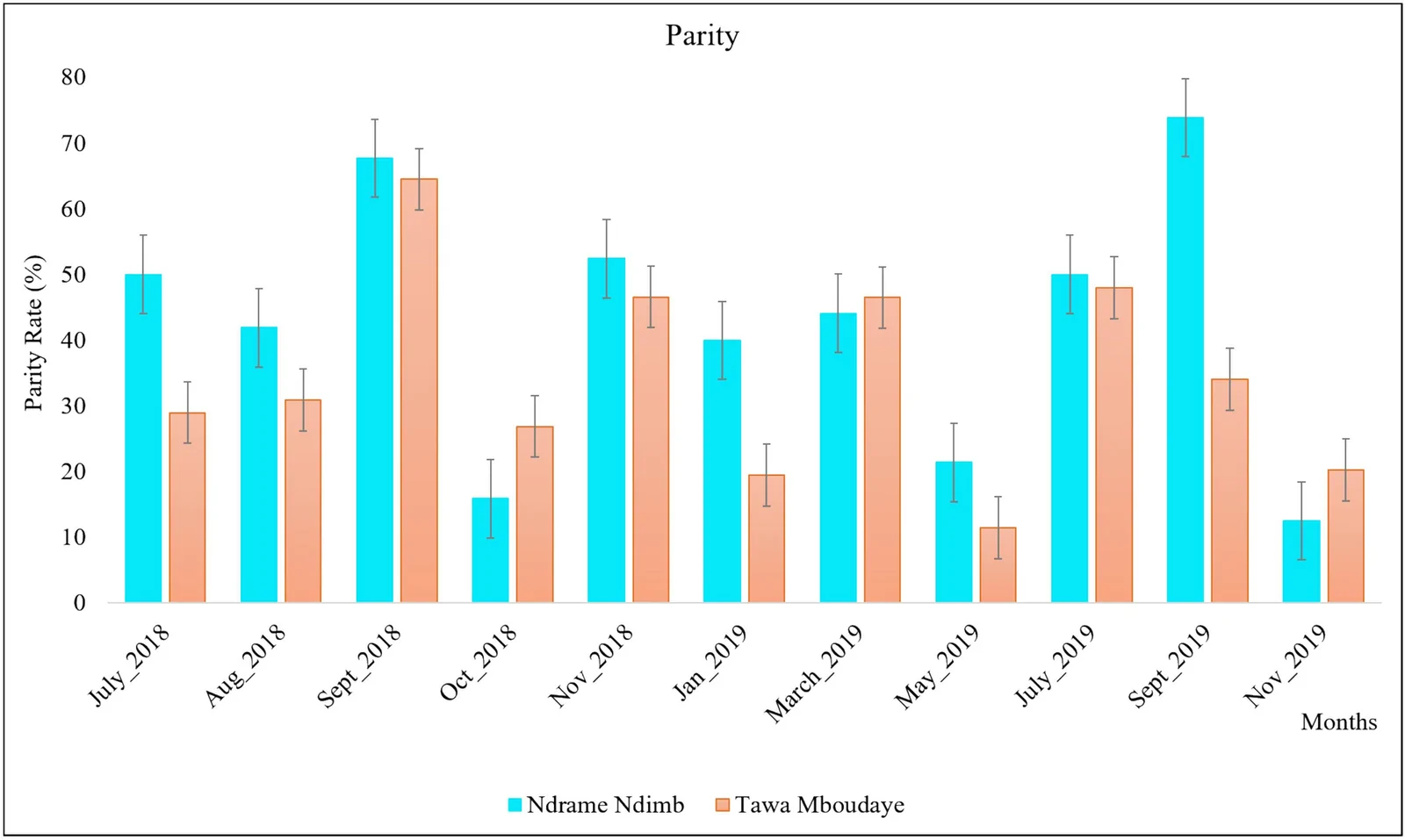
Parity rate of *Anopheles funestus* s.s in Tawa Mboudaye and Ndramé Ndimb

The overall parity rate of *An. funestus* s.s. was 45.7% in Ndramé Ndimb and 38.3% in Tawa Mboudaye. This difference in longevity between the two sites was statistically significant (*χ*^2^ = 138.4, *df* = 1, *p* < 0.001). Across all sites, the highest parity rates were recorded in the rainy season (September) for the two survey years, with 64.5% of parous females in Tawa Mboudaye (2018) and 67.7% and 73.9% in Ndramé Ndimb in 2018 and 2019, respectively (Fig. 3).

### *Plasmodium falciparum* infection rates and EIR

A total of 1,973 *An. funestus* s.s females were tested for the presence of *P. falciparum* circumsporozoite proteins by ELISA. Six *An. funestus* s.s. specimens were found to be infected with *P. falciparum,* corresponding to an infection rate of 0.3%. In Ndramé Ndimb, the infection rate was 0.3% (1/363) (CI_95%_: 0.0—1.5%), while in Tawa Mboudaye, it was 0.31% (5/1,615) (CI_95%_: 0.10—0.72%). No significant difference in infection rates was observed between the two sites (*χ* ^2^ = 0.0116, df = 1, *p* = 0.913).

The mean entomological inoculation rate (EIR) was 0.023 infected bites per person per night (ib/p/n), corresponding to an annual EIR of 8.40 infected bites per person per year. Throughout the study period, EIRs remained low in both sites, with values of 0.008 ib/p/n in Ndramé Ndimb and 0.038 ib/p/n in Tawa Mboudaye, corresponding to an annual EIRs of 2.92 and 13.87 infected bites per person per year, respectively. In Tawa Mboudaye, 80% (5/6) of infected mosquitoes were collected during the rainy season, notably in July (n = 1), August (n = 2) and September (n = 1), but also during the dry season in November (n = 1) (Table 5). On the other hand, most transmission (60%; 3/5) in Tawa Mboudaye occurred outdoors during the second half of the night, between 2 a.m. and 5 a.m. Conversely, in Ndramé Ndimb, infected mosquitoes were collected during the dry season (May), with transmission occurring indoors, between 3 a.m. and 4 a.m.

**Table 5:** Seasonal variations of *P. falciparum* infection and entomological inoculation rates among *Anopheles funestus* s.s females caught by HLC

Mont—Year	Ndramé Ndimb					Tawa Mboudaye					Total				
	Tested	Positive	CSPR (%)	HBR	EIR (ib/p/n)	Tested	Positive	CSPR (%)	HBR	EIR (ib/p/n)	Tested	Positive	CSPR (%)	HBR	EIR (ib/p/n)
Jul_2018	23	0	0,000	2,000	0,000	145	1	0,007	12,167	0,084	168	1	0,006	7,083	0,042
Aug_2018	47	0	0,000	3,917	0,000	324	2	0,006	27,083	0,167	371	2	0,005	15,500	0,084
Sept_2018	62	0	0,000	5,250	0,000	178	1	0,006	14,833	0,083	240	1	0,004	10,042	0,042
Oct_2018	46	0	0,000	3,833	0,000	134	0	0,000	11,583	0,000	180	0	0,000	7,708	0,000
Nov_2018	26	0	0,000	2,167	0,000	78	0	0,000	6,667	0,000	104	0	0,000	4,417	0,000
Jan_2019	31	0	0,000	2,583	0,000	98	0	0,000	8,417	0,000	129	0	0,000	5,500	0,000
Marc_2019	69	0	0,000	5,750	0,000	223	0	0,000	18,917	0,000	292	0	0,000	12,333	0,000
May_2019	22	1	0,045	1,917	0,087	31	0	0,000	2,750	0,000	53	1	0,019	2,333	0,044
Jul_2019	11	0	0,000	0,917	0,000	136	0	0,000	11,417	0,000	147	0	0,000	6,167	0,000
Sept_2019	26	0	0,000	2,167	0,000	203	0	0,000	17,250	0,000	229	0	0,000	9,708	0,000
Nov_2019	0	0	0,000	0,833	0,000	65	1	0,015	5,667	0,087	65	1	0,015	3,250	0,050
Total	363	1	0,003	2,848	0,008	1615	5	0,003	12,432	0,038	1978	6	0,003	7,640	0,023

CSPR: Circumsporozoite Infection Rate, EIR: Entomological Inoculation Rate, ib/p/n: infected bites per person per night

## Discussion

This study aimed to assess the contribution of *An. funestus* s.s in malaria transmission in the central Senegal. Seven *Anopheles* species, previously documented in Senegal [22], were collected, indicating high species diversity. This diversity may be attributed to the variety of larval breeding sites across the study area, as observed in Central and West Africa. The relatively high abundance of *An. funestus* s.s in the study sites is also in line with the presence of potential breeding sites of this species [29], which have the same hydro-geographical characteristics as the northern zone, which in Senegal is the predilection zone for this species, in particular Lac de Guiers area and Senegal River basin [30, 31].

In Tawa Mboudaye, the density of *An. funestus* s.s was relatively higher compared to Ndramé Ndimb. This difference could be attributed to the presence of stable breeding sites in Tawa Mboudaye, which are suitable for *An. funestus* s.s larval development*,* known to be stable permanent or semi-permanent water bodies with emergent or floating aquatic vegetation [32–34].

*Anopheles funestus* s.s displayed a tendency to bite predominantly during the second half of the night, which may reduce its potential for residual malaria transmission, as people are generally asleep and protected by bed nets until 6 a.m. This species is widely distributed in rural areas of Senegal and may maintain low levels of malaria transmission [13, 16, 35]. Notably, *An. funestus* s.s was found to bite more frequently outdoors, contrasting with observations in Cameroon, where it predominantly bites indoors [33]. Similar outdoor biting behaviour had also been observed previously in Dielmo [22, 36]. High human-biting rates recorded during both the rainy and dry seasons in Tawa Mboudaye were also reported in the Guiers Lake area [16]. The low anthropophilic rates observed in this study are unusual for *An. funestus* s.s*,* a species generally considered as highly anthropophilic malaria vector. However, in our study, *Anopheles funestus* s.s populations displayed more zoophilic tendencies, possibly due to the presence and accessibility of animals in contrast to the human host which has probably become unavailable though the use of mosquito bed nets [21]. This shift in biting behaviour contrasts with studies carried out in Cameroon [37, 38], Kenya [34], Ghana [39] and Ethiopia [40, 41], where *An. funestus* s.s was found to be predominantly anthropophilic. Moreover, the notable reduction in human biting rates in 2019 coincided with the mass distribution of LLINs by the NMCP all over the country, including the study area. Long-lasting Insecticidal Nets represent the primary protection tool at the community-level, reducing human-vector contact and their widespread use may induce exophilic behaviour in mosquitoes thus diverted to most available animal alternative due to the deterrent, repellent and lethal effects of pyrethroids nets [42].

In our study area, *An. funestus* s.s populations appeared relatively young, with low parity rates observed in both Ndramé Ndimb and Tawa Mboudaye. This finding is unusual for *An. funestus* s.s, as its female longevity typically contributes to its role as a major malaria vector in Senegal [13, 16] and across Africa [43, 44]. The decline in HBR and the low anthropophilic rates observed are likely linked to intensified vector control efforts, which may also reduce mosquito vector longevity and induce behavioural shifts, ultimately lowering malaria infection risk [45]. The infection rate observed in this study was lower than those generally recorded for *An. funestus* s.s in Senegal [16, 20, 36, 46] and across Africa [47, 48]. This low infection rate may be associated with the increased zoophilic and exophilic tendencies of the local *An. funestus* s.s population. Similar behavioural changes have been observed in Tanzania, where *An. funestus* s.s populations exhibited strong exophilic and zoophilic behaviour after LLINs implementation [49].

Nevertheless, despite the low infection rate, *An. funestus* s.s females were found infected with *Plasmodium falciparum*, confirming their role in malaria transmission in central Senegal. Transmission risk is particularly high outdoors during the second half of the night, and populations may face increased exposure if vector control measures are not used consistently, especially during periods of heightened outdoor activity due to elevated temperatures and sensation of heat inducing people to stay longer and even sleep outdoor (personal observation). In Senegal and across sub-Saharan Africa, LLINs and IRS are the main malaria vector control strategies [50], aiming to reduce human-vector contact and vector longevity. Previous studies have documented the contribution of *An. funestus* s.s populations to malaria transmission in Senegal [12, 22, 36, 46]. The low transmission levels observed in this study may reflect the effectiveness of these control strategies. However, while findings from two specific districts provide important evidence of the persistence of *An. funestus* s.s, the low infection rates and high zoophily observed reveal a complex transmission landscape that may not be fully representative of all central Senegal. These results underscore the role of *An. funestus* s.s in sustaining residual malaria transmission and highlight the importance of considering its behavioural plasticity in vector control efforts.

## Conclusions

This study highlights the predominance of *Anopheles funestus* s.s. and its important role in sustaining residual malaria transmission in central Senegal, even where overall transmission intensity remains low. The observed high zoophily and persistent activity of this species provide valuable evidence to support the National Malaria Control Programme (NMCP) in refining vector control strategies beyond traditional indoor measures. Further research on the distribution of *An. funestus* across central Senegal is needed to identify breeding sites and residual transmission hotspots, as well as to assess insecticide susceptibility and behavioural resilience. A thorough understanding of the bionomics of *An. funestus* will help guide the selection of tailored vector control measures to support the NMCP’s malaria elimination goals.

## Data Availability

No datasets were generated or analysed during the current study.

## Abbreviations

LLIN: Long-lasting Insecticidal Net
HLC: Human landing catch
PSC: Pyrethrum spray catch
IRD: Indoor resting density
HBR: Human biting rate
HBI: Human blood index
ELISA: Enzyme-linked immunosorbent assay
CSPR: Circumsporozoite infection rates
EIR: Entomological inoculation rates
IRS: Indoor residual spraying

## References

[1] WHO. World Malaria Report 2025. Wordl Health Organization. 2025.

[2] WHO. World Malaria Report 2021. Wordl Health Organization. 2021.

[3] WHO.. World malaria report 2020. Wordl Health Organization. 2020.

[4] PNLP. Programme National de Lutte contre le Paludisme Bulletin Epidemiologique Annuel 2024.

[5] Sambe BS, Ousmane S, Diaw M, Diagne A, Sab A, Senghor B, et al. Common Molecular Detection of the Neglected Human Malaria Parasite Among Febrile Patients in Southern Regions in Senegal. 2025;1–11. 10.3390/pathogens14121201 PMC12735732 41471157

[6] Sinka ME, Pironon S, Massey NC, Longbottom J, Hemingway J, Moyes CL, et al. A new malaria vector in Africa: predicting the expansion range of *Anopheles stephensi* and identifying the urban populations at risk. Proc Natl Acad Sci USA. 2020;117(40):24900–8. 10.1073/pnas.2003976117 PMC7547157 32929020

[7] Afrane YA, Bonizzoni M, Yan G. Secondary Malaria Vectors of Sub-Saharan Africa: Threat to Malaria Elimination on the Continent? Current Topics in Malaria. 2016;(November).

[8] Noor AM, Kinyoki DK, Mundia CW, Kabaria CW, Mutua JW, Alegana VA, et al. The changing risk of Plasmodium falciparum malaria infection in Africa: 2000–10: A spatial and temporal analysis of transmission intensity. Lancet. 2014;383(9930):1739–47. 10.1016/S0140-6736(13)62566-0 PMC4030588 24559537

[9] Robert V, Macintyre K, Keating J, Trape JF, Duchemin JB, Warren M, et al. Malaria transmission in urban sub-Saharan Africa. Am J Trop Med Hyg. 2003;68(2):169–76. 12641407

[10] Belisse D, Belisse PD, Kopya E, Ngadjeu CS, Chiana NS, Talipouo A, et al. Urban malaria in sub ‑ Saharan Africa : dynamic of the vectorial system and the entomological inoculation rate. Malaria Journal. 2021;1–18. 10.1186/s12936-021-03891-z PMC8424958 34493280

[11] Selvaraj P, Wenger EA, Gerardin J. Seasonality and heterogeneity of malaria transmission determine success of interventions in high-endemic settings: A modeling study. BMC Infect Dis. 2018;18(1):1–14. 10.1186/s12879-018-3319-y PMC6104018 30134861

[12] PNLP. Programme National de Lutte contre le Paludisme Bulletin Epidémiologique annuel 2021. 2021;

[13] Fontenille D, Lochouarn L. The of the malaria vectorial system in Africa. 1999;267–71. 10697867

[14] Konate L, Diagne N, Brahimi K, Faye O, Legros F, Rogier C, et al. Biologie des vecteurs et transmission de plasmodium falciparum, p. malariae et p. ovale dans un village de savane d’afrique de l’ouest (Dielmo, Sénégal) . Parasite. 1994;1(4):325–33. 10.1051/parasite/1994014325 9235206

[15] Coetzee M, Craig M, Le Sueur D. Distribution of African malaria mosquitoes belonging to the *Anopheles gambiae* complex. Parasitol Today. 2000;16(2):74–7. 10.1016/s0169-4758(99)01563-x 10652493

[16] Dia I, Konate L, Samb B, Sarr JB, Diop A, Rogerie F, et al. Bionomics of malaria vectors and relationship with malaria transmission and epidemiology in three physiographic zones in the Senegal River Basin. Acta Trop. 2008. 10.1016/j.actatropica.2007.10.010. 18068685

[17] Sy O, Sarr PC, Assogba BS, Nourdine MA, Ndiaye A, Konaté L, et al. Residual malaria transmission and the role of Anopheles arabiensis and Anopheles melas in central Senegal. J Med Entomol. 2023;60(3):546–53. 10.1093/jme/tjad020 PMC10179433 36932704

[18] Vezenegho SB, Chiphwanya J, Hunt RH, Coetzee M, Bass C, Koekemoer LL. Characterization of the *Anopheles funestus* group, including *Anopheles funestus*-like, from Northern Malawi. Trans R Soc Trop Med Hyg. 2013. 10.1093/trstmh/trt089. 24189481

[19] Fontenille D. ELISA detection of malaria sporozoites : false-positive results in Anopheles gambiae s . l . associated with bovine bloodmeals. 1995;101–2. 10.1016/s0035-9203(99)90198-0 10492803

[20] Dia I, Lochouarn L, Diatta M, Sokhna CS, Fontenille D. P références trophiques des femelles endophiles d ’ Anopheles funestus au Sénégal. Entomologie médicale. 2000;1:210–3. 16579080

[21] Gueye A, Hadji E, Ngom M, Diagne A, Ndoye BB, Dione ML, et al. Host feeding preferences of malaria vectors in an area of low malaria transmission To cite this version : Host feeding preferences of malaria vectors in an area of low malaria transmission. Scientific Reports. 2023;0–10. 10.1038/s41598-023-43761-z PMC10542387 37775717

[22] Diagne N, Fontenille D, Konate L, Faye O, Lamizana MT, Legros F, et al. Anopheles of Senegal. An annotated and illustrated list. Bulletin de la Société de Pathologie Exotique. 1994;87(4):267–77. 7866049

[23] Coetzee M. Key to the females of Afrotropical Anopheles mosquitoes (Diptera: Culicidae). Malar J. 2020;19(1):1–20. 10.1186/s12936-020-3144-9 PMC7020601 32054502

[24] Detinova TS, Gillies MT. Observations on the Determination of the Age Composition and Epidemiological Importance of Populations of Anopheles gambiae Giles and Anopheles funestus Giles in Tanganyika. Bulletin of Wordl Health Organization. 1964;23–8. PMC2554915 14122438

[25] Koekemoer LL, Kamau L, Hunt RH, Coetzee M. A cocktail polymerase chain reaction assay to identify members of the Anopheles funestus (Diptera: Culicidae) group. Am J Trop Med Hyg. 2002;66(6):804–11. 10.4269/ajtmh.2002.66.804 12224596

[26] Wirtz RA, Zavala F, Charoenvit Y, Campbell GH, Burkot TR, Schneider I, et al. Comparative testing of monoclonal antibodies against Plasmodium falciparum sporozoites for ELISA development. Bulletin of World Health Organization. 1987;65(1):39–45. PMC2490858 3555879

[27] Beier JC, Perkins PV, Wirtz RA, Koros J, Diggs D, Gargan TP, et al. Bloodmeal identification by direct enzyme-linked immunosorbent assay (ELISA), tested on Anopheles (Diptera: Culicidae) in Kenya. J Med Entomol. 1988;25(1):9–16. 10.1093/jmedent/25.1.9 3357176

[28] Doyle, J.J.; Doyle JL. A rapid DNA isolation procedure for small quantities of fresh leaf tissue. Phytochimical bulletin; 1987. p. 11–5.

[29] Ayala D, Costantini C, Ose K, Kamdem GC, Antonio-Nkondjio C, Agbor JP, et al. Habitat suitability and ecological niche profile of major malaria vectors in Cameroon. Malar J. 2009;8(1):1–15. 10.1186/1475-2875-8-307 PMC2805691 20028559

[30] Konate L, Diop A, Sy N, Faye MN, Deng Y, Izri A, et al. Comeback of Anopheles funestus in Sahelian Senegal [11]. Lancet. 2001;358(9278):336. 10.1016/s0140-6736(01)05517-9 11511072

[31] Samb B, Konate L, Irving H, Riveron JM, Dia I, Faye O, et al. Investigating molecular basis of lambda-cyhalothrin resistance in an Anopheles funestus population from Senegal. Parasit Vectors. 2016;9(1):1–10. 10.1186/s13071-016-1735-7 PMC4983014 27519696

[32] Gillies M.T. and Coetzee M. A supplement to the Anophelinae of Africa South of the Sahara. South African Institute Medical Research. 1987;55(55).

[33] Djamouko-djonkam L, Nkahe DL, Kopya E, Talipouo A. Implication d ’ Anopheles funestus dans la transmission du paludisme dans la ville de Yaoundé , Cameroun. Parasite. 2020; 10.1051/parasite/2020005 PMC7015064 32048986

[34] Debrah I, Afrane YA, Amoah LE, Ochwedo KO, Mukabana WR, Zhong D, et al. Larval ecology and bionomics of *Anopheles funestus* in highland and lowland sites in western Kenya. PLoS ONE. 2021;16(10 October):1–20. 10.1371/journal.pone.0255321 PMC8504749 34634069

[35] Wangrawa DW, Odero JO, Baldini F, Okumu F, Badolo A. Distribution and insecticide resistance profile of the major malaria vector *Anopheles funestus* group across the African continent. Med Vet Entomol. 2024;38(2):119–37. 10.1111/mve.12706 38303659

[36] Doucoure S, Thiaw O, Thiaw O, Wotodjo AN, Bouganali C, Diagne N, et al. Anopheles arabiensis and Anopheles funestus biting patterns in Dielmo, an area of low level exposure to malaria vectors. Malaria Journal. 2020;19(1). 10.1186/s12936-020-03302-9 PMC7320554 32590996

[37] Tabue RN, Awono-Ambene P, Etang J, Atangana J, Antonio-Nkondjio C, Toto JC, et al. Role of Anopheles (Cellia) rufipes (Gough, 1910) and other local anophelines in human malaria transmission in the northern savannah of Cameroon: a cross-sectional survey. Parasites and Vectors. 2017;10(1). 10.1186/s13071-016-1933-3 PMC5225577 28077167

[38] Ekoko WE, Awono-Ambene P, Bigoga J, Mandeng S, Piameu M, Nvondo N, et al. Patterns of anopheline feeding/resting behaviour and Plasmodium infections in North Cameroon, 2011–2014: Implications for malaria control. Parasit Vectors. 2019;12(1):1–12. 10.1186/s13071-019-3552-2 PMC6567421 31196161

[39] Dadzie SK, Brenyah R, Appawu MA. Role of species composition in malaria transmission by the Anopheles funestus group ( Diptera : Culicidae ) in Ghana. J Vector Ecol. 2012;38(1):105–10. 10.1111/j.1948-7134.2013.12015.x 23701614

[40] Adugna T, Yewhelew D, Getu E. Bloodmeal sources and feeding behavior of anopheline mosquitoes in Bure district, northwestern Ethiopia. Parasit Vectors. 2021;14(1):1–12. 10.1186/s13071-021-04669-7 PMC7977575 33741078

[41] Massebo F, Balkew M, Gebre-Michael T, Lindtjørn B. Blood meal origins and insecticide susceptibility of Anopheles arabiensis from Chano in South-West Ethiopia. Parasit Vectors. 2013;6(1):1–10. 10.1186/1756-3305-6-44 PMC3606335 23433306

[42] Miller JR, Siegert PY, Amimo FA, Walker ED. Designation of chemicals in terms of the locomotor responses they elicit from insects: an update of Dethier et al. (1960). J Econ Entomol. 1960;102(6):2056–60. 10.1603/029.102.0606 20069831

[43] Mendis C, Jacobsen JL, Gamage-Mendis A, Bule E, Dgedge M, Thompson R, et al. *Anopheles arabiensis* and *An. funestus* are equally important vectors of malaria in Matola coastal suburb of Maputo, southern Mozambique. Med Vet Entomol. 2000. 10.1046/j.1365-2915.2000.00228.x. 10872861

[44] Takken W, Charlwood JD, Billingsley PF, Gort G. Dispersal and survival of *Anopheles funestus* and *A. gambiae* s.l. (Diptera: Culicidae) during the rainy season in southeast Tanzania. Bull Entomol Res. 1998. 10.1017/s0007485300026080.

[45] World malaria report 2018. Wordl Health Organization 2018.

[46] Dia I, Diop T, Rakotoarivony I, Kengne P. Bionomics of Anopheles gambiae Giles , An . arabiensis Patton , An . funestus Giles and An . nili ( Theobald ) ( Diptera : Culicidae ) and Transmission of Plasmodium falciparum in a Sudano-Guinean Zone ( Ngari , Senegal ). Journal of Medical Entomology. 2003;279–83. 10.1603/0022-2585-40.3.279 12943105

[47] Fontenille D, Lochouarn L, Sokhna C. High annual and seasonal variations in malaria transmission anophelines and vector species composition in dielmo, a holoendemic area in senegal. Am J Trop Med Hyg. 1997;56(3):247–53. 10.4269/ajtmh.1997.56.247 9129525

[48] Coetzee M, Fontenille D. Advances in the study of Anopheles funestus, a major vector of malaria in Africa. In: Insect Biochemistry and Molecular Biology. 2004. 10.1016/j.ibmb.2004.03.012 15242700

[49] Russell TL, Govella NJ, Azizi S, Drakeley CJ, Kachur SP, Killeen GF. Increased proportions of outdoor feeding among residual malaria vector populations following increased use of insecticide-treated nets in rural Tanzania. Malar J. 2011;10:1–10. 10.1186/1475-2875-10-80 PMC3084176 21477321

[50] PMI. Rapport d’évaluation du système de surveillance, suivi, évaluation du paludisme au Sénégal. 2020;

